# Supplementary material for: The Effect of (Poly)phenol-Rich Interventions on Cognitive Functions and Neuroprotective Measures in Healthy Aging Adults: A Systematic Review and Meta-Analysis
Source: J Clin Med. 2020 Mar 19;9(3):835. doi: 10.3390/jcm9030835 (PMC7141326; doi:10.3390/jcm9030835)
Supplement: Supplementary file 1 [file jcm-09-00835-s001.pdf]

Appendix S1.

Search Strategy

Search terms (including mapping to appropriate MeSH terms where appropriate) described major (poly)phenol classes (polyphenol OR flavonoids OR polyphenolic compounds OR isoflavone OR flavanol OR phytoestrogen OR resveratrol) in combination with keywords relating to cognitive and brain functions (cognitive performance OR cognitive OR cognitive function OR cognition OR brain function OR executive function OR attention OR working memory OR brain imaging OR neuroimaging OR neural OR magnetic resonance imaging OR MRI OR fMRI OR grey matter OR gray matter OR brain volume OR brain structure OR electrophysiology OR EEG OR event related potential OR neuroblast OR neuroblast OR cerebral blood flow OR CBF OR brain-derived neurotrophic factor OR BDNF OR regional perfusion OR cerebrovascular responsiveness OR CVR OR Pulsatility index OR transcranial doppler OR TCD OR Near-Infrared Spectroscopy OR NIRS OR cerebral hemodynamics OR total hemoglobin OR total-Hb OR oxygenated hemoglobin OR oxy-Hb OR deoxygenated hemoglobin OR deoxy-Hb). The search was conducted with additional filters excluding not human studies (NOT (mice OR rats OR animals)) and studies in diseases (NOT (Parkinson's OR stroke OR Alzheimer's OR dementia OR cancer OR lesions OR diabetes OR injury OR patients OR disease OR impairment)).

Separate Pubmed and Web of Science search strategies

Pubmed:

(((((((polyphenol) OR flavonoids) OR polyphenolic compounds) OR isoflavone) OR flavanol) OR phytoestrogen) OR resveratrol) AND (((((((((((((((((((((((((((((((((((((((cognitive performance) OR cognitive) OR cognitive function) OR cognition) OR brain function) OR executive function) OR attention) OR working memory) OR brain imaging) OR neuroimaging) OR neural) OR magnetic resonance imaging) OR MRI) OR fmri) OR grey matter) OR gray matter) OR brain volume) OR brain structure) OR electrophysiology) OR EEG) OR event related potential OR neuroblast) OR neuroblast) OR cerebral blood flow) OR CBF) OR regional perfusion) OR brain-derived neurotrophic factor) OR BDNF) OR cerebrovascular responsiveness) OR CVR) OR Pulsatility index) OR transcranial doppler) OR TCD) OR Near-Infrared Spectroscopy) OR NIRS) OR cerebral hemodynamics) OR total hemoglobin) OR total-Hb) OR oxygenated hemoglobin) OR oxy-Hb) OR deoxygenated

hemoglobin) OR deoxy-Hb) NOT ((((((((((((((mice) OR animals) OR Parkinson's) OR stroke) OR Alzheimer’s) OR dementia) OR cancer) OR lesions) OR diabetes) OR injury) OR patients) OR rats) OR disease) OR impairment)

Web of science

TS = (polyphenol OR flavonoids OR polyphenolic compounds OR isoflavone OR flavanol OR phytoestrogen OR resveratrol) AND TS = (cognitive performance OR cognitive OR cognitive function OR cognition OR brain function OR executive function OR attention OR working memory OR brain imaging OR neuroimaging OR neural OR magnetic resonance imaging OR MRI OR fmri OR grey matter OR gray matter OR brain volume OR brain structure OR electrophysiology OR EEG OR event related potential OR neuroblast OR neuroblast OR cerebral blood flow OR CBF OR brain-derived neurotrophic factor OR BDNF OR regional perfusion OR cerebrovascular responsiveness OR CVR OR Pulsatility index OR transcranial doppler OR TCD OR Near-Infrared Spectroscopy OR NIRS OR cerebral hemodynamics OR total hemoglobin OR total-Hb OR oxygenated hemoglobin OR oxy-Hb OR deoxygenated hemoglobin OR deoxy-Hb) NOT TS = (mice OR rats OR animals OR Parkinsons OR stroke OR Alzheimer OR dementia OR cancer OR lesions OR diabetes OR injury OR patients OR disease OR impairment OR)

Appendix S2. PRISMA checklist

Section/topic	#	Checklist item	Reported on page #
**TITLE**			
Title	1	Identify the report as a systematic review, meta-analysis, or both.	1
**ABSTRACT**			
Structured summary	2	Provide a structured summary including, as applicable, background; objectives; data sources; study eligibility criteria, participants, and interventions; study appraisal and synthesis methods; results; limitations; conclusions and implications of key findings; and systematic review registration number.	1,2
**INTRODUCTION**			
Rationale	3	Describe the rationale for the review in the context of what is already known.	2,3
Objectives	4	Provide an explicit statement of questions being addressed with reference to participants, interventions, comparisons, outcomes, and study design (PICOS).	3
**METHODS**			
Protocol and registration	5	Indicate if a review protocol exists, and if and where it can be accessed (e.g., Web address), and if available, provide registration information including registration number.	3,4
Eligibility criteria	6	Specify study characteristics (e.g., PICOS and length of follow-up) and report characteristics (e.g., years considered, language, and publication status) used as criteria for eligibility, giving rationale.	3,4
Information sources	7	Describe all information sources (e.g., databases with dates of coverage and contact with study authors to identify additional studies) in the search and date last searched.	3-5
Search	8	Present full electronic search strategy for at least one database, including any limits used, such that it could be repeated.	3
Study selection	9	State the process for selecting studies (i.e., screening, eligibility, inclusion in the systematic review, and if applicable inclusion in the meta-analysis).	3–5
Data collection process	10	Describe method of data extraction from reports (e.g., piloted forms, independently, and in duplicate) and any processes for obtaining and confirming data from investigators.	5
Data items	11	List and define all variables for which data were sought (e.g., PICOS and funding sources) and any assumptions and simplifications made.	4
Risk of bias in individual studies	12	Describe methods used for assessing risk of bias of individual studies (including specification of whether this was done at the study or outcome level) and how this information is to be used in any data synthesis.	14
Summary measures	13	State the principal summary measures (e.g., risk ratio, and difference in means).	14
Synthesis of results	14	Describe the methods of handling data and combining results of studies, if done, including measures of consistency (e.g., I^2) for each meta-analysis.	14

Section/topic	#	Checklist item	Reported on page #
Risk of bias across studies	15	Specify any assessment of risk of bias that may affect the cumulative evidence (e.g., publication bias and selective reporting within studies).	14
Additional analyses	16	Describe methods of additional analyses (e.g., sensitivity or subgroup analyses and meta-regression), if done, indicating which were prespecified.	14
**RESULTS**			
Study selection	17	Give numbers of studies screened, assessed for eligibility, and included in the review, with reasons for exclusions at each stage, ideally with a flow diagram.	15
Study characteristics	18	For each study, present characteristics for which data were extracted (e.g., study size, PICOS, and follow-up period) and provide the citations.	15,16
Risk of bias within studies	19	Present data on risk of bias of each study and, if available, any outcome level assessment (see item 12).	19–25
Results of individual studies	20	For all outcomes considered (benefits or harms), present for each study (a) simple summary data for each intervention group and (b) effect estimates and confidence intervals, ideally with a forest plot.	19–25
Synthesis of results	21	Present results of each meta-analysis done, including confidence intervals and measures of consistency.	20–25
Risk of bias across studies	22	Present results of any assessment of risk of bias across studies (see Item 15).	20–25
Additional analysis	23	Give results of additional analyses, if done (e.g., sensitivity or subgroup analyses and meta-regression (see Item 16)).	25
**DISCUSSION**			
Summary of evidence	24	Summarize the main findings including the strength of evidence for each main outcome; consider their relevance to key groups (e.g., healthcare providers, users, and policy makers).	25–27
Limitations	25	Discuss limitations at study and outcome level (e.g., risk of bias) and at the review-level (e.g., incomplete retrieval of identified research, and reporting bias).	27
Conclusions	26	Provide a general interpretation of the results in the context of other evidence and implications for future research.	27
**FUNDING**			
Funding	27	Describe sources of funding for the systematic review and other support (e.g., supply of data) and role of funders for the systematic review.	28

From: Moher D, Liberati A, Tetzlaff J, Altman DG, The PRISMA Group (2009). Preferred Reporting Items for Systematic Reviews and Meta-Analyses: The PRISMA Statement. PLoS Med 6(6): e1000097. doi:10.1371/journal.pmed1000097

For more information, visit: **www.prisma-statement.org**.
